# The cognitive and perceptual correlates of ideological attitudes: a data-driven approach

**DOI:** 10.1098/rstb.2020.0424

**Published:** 2021-04-12

**Authors:** Leor Zmigrod, Ian W. Eisenberg, Patrick G. Bissett, Trevor W. Robbins, Russell A. Poldrack

**Affiliations:** ^1^ Department of Psychology, University of Cambridge, Cambridge, UK; ^2^ Behavioural and Clinical Neuroscience Institute, University of Cambridge, Cambridge, UK; ^3^ Department of Psychology, Stanford University, Stanford, CA 94305, USA

**Keywords:** ideological cognition, political psychology, perception, drift-diffusion model, attitudes, dogmatism

## Abstract

Although human existence is enveloped by ideologies, remarkably little is understood about the relationships between ideological attitudes and psychological traits. Even less is known about how cognitive dispositions—individual differences in how information is perceived and processed— sculpt individuals' ideological worldviews, proclivities for extremist beliefs and resistance (or receptivity) to evidence. Using an unprecedented number of cognitive tasks (*n* = 37) and personality surveys (*n* = 22), along with data-driven analyses including drift-diffusion and Bayesian modelling, we uncovered the specific psychological signatures of political, nationalistic, religious and dogmatic beliefs. Cognitive and personality assessments consistently outperformed demographic predictors in accounting for individual differences in ideological preferences by 4 to 15-fold. Furthermore, data-driven analyses revealed that individuals’ ideological attitudes mirrored their cognitive decision-making strategies. Conservatism and nationalism were related to greater caution in perceptual decision-making tasks and to reduced strategic information processing, while dogmatism was associated with slower evidence accumulation and impulsive tendencies. Religiosity was implicated in heightened agreeableness and risk perception. Extreme pro-group attitudes, including violence endorsement against outgroups, were linked to poorer working memory, slower perceptual strategies, and tendencies towards impulsivity and sensation-seeking—reflecting overlaps with the psychological profiles of conservatism and dogmatism. Cognitive and personality signatures were also generated for ideologies such as authoritarianism, system justification, social dominance orientation, patriotism and receptivity to evidence or alternative viewpoints; elucidating their underpinnings and highlighting avenues for future research. Together these findings suggest that ideological worldviews may be reflective of low-level perceptual and cognitive functions.

This article is part of the theme issue ‘The political brain: neurocognitive and computational mechanisms’.

## 1. Introduction

One of the most powerful metaphors in political psychology has been that of *elective affinities*—the notion that there is a mutual attraction between ‘the structure and contents of belief systems and the underlying needs and motives of individuals and groups who subscribe to them’ [1]. With roots in Enlightenment philosophy and Max Weber's sociology, this metaphor contends that certain ideologies resonate with the psychological predispositions of certain people. So, we can elucidate psycho-political processes by logically tracing these coherences, these elective affinities between ideas and interests. This analogy has inspired rich theories about the epistemic, relational and existential motivations that drive individuals to adhere to political ideologies (e.g. [2]), highlighting the role of needs for coherence, connectedness and certainty in structuring ideological attitudes (e.g. [3–5]).

Nonetheless, the methodologies employed to study these questions have been mostly of a social psychological nature, relying primarily on self-report measures of needs for order, cognitive closure, rigidity and others (e.g. [2]). This has skewed the academic conversation towards the *needs* and *interests* that ideologies satisfy, and obscured the role of *cognitive dispositions* that can promote (or suppress) ideological thinking [6]. In fact, it is only recently that researchers have begun to employ neurocognitive tasks and analytic approaches from cognitive science in order to tackle the question: *which cognitive traits shape an individual's ideological worldviews*? In this investigation, we sought to apply cognitive methodologies and analytic tools in order to identify the cognitive and personality correlates of ideological attitudes in a data-driven fashion. Borrowing methods from cognitive psychology, which have established sophisticated techniques to measure and analyse perceptual and cognitive processes in an objective and implicit way, and implementing these in the study of ideology can facilitate the construction of a more wholistic and rigorous cognitive science of ideology. This can push the analogy of ‘elective affinities’ into the realm of perception and cognition to allow us to tackle the question: are there parallels between individuals' ideologies and their general perceptual or cognitive styles and strategies?

Furthermore, owing to limited resources and siloed research disciplines, many studies in social psychology frequently focus on a single ideological domain (e.g. political conservatism) or a single psychological domain (e.g. analytical thinking). While an in-depth focus on a specific domain is essential for theoretical development, the selection of hypotheses and methodologies can at times suffer from problems of bias and a lack of conceptual integration across different ideological and psychological domains. Indeed, a growing concern has emerged among researchers that psychologists of politics, nationalism and religion generate hypotheses and develop study designs that confirm their prior beliefs about the origins of social discord [7–12]. It is, therefore, valuable to complement theory-driven research with data-driven approaches, which can help to overcome these methodological challenges, as well as offer a wholistic view of these complex relationships by ‘letting the data speak’. Perhaps most importantly, data-driven research can help validate or challenge theory-driven findings and consequently offer directions for future research.

The present investigation, therefore, aimed to harness novel cognitive approaches, a data-driven study design, a mix of frequentist and Bayesian analytic approaches and a wide-ranging assessment of both psychological traits and ideological domains. It was motivated by the questions: to what extent do the ideologies people espouse reflect their cognitive and personality characteristics? What are the commonalities and differences between the psychological underpinnings of diverse ideological orientations? What are the contributions of cognitive processes versus personality traits to the understanding of ideologies? and which psychological traits are associated with one's likelihood of being attracted to particular ideologies?

Importantly, although a rigorous cognitive science of ideology may be at its infancy, these questions are not entirely new—scholars across the sciences and humanities have long theorized about the psychological origins of citizens' political, nationalistic and religious attitudes [2,13]. A fertile literature has revealed that individuals’ ideological inclinations are related to various psychological traits, such as their personal needs for order and structure [3–5], cognitive flexibility [6,14–18], metacognition and learning styles [19,20] and even perceptual reactivity to negative information [21–24]. The advent of political neuroscience [25], illustrating the neural structures and processes that underpin (political) ideology [26–32], spurs even more profound questions about the ways in which cognitive mechanisms may mediate between the brain and belief.

Ideologies can be generally described as doctrines that rigidly prescribe epistemic and relational norms or forms of hostility [33]. The present investigation espouses a domain-general outlook towards the definition of ideology—focusing on the factors associated with thinking ideologically in multiple domains, such as politics, nationalism and religion. This includes dogmatism, which can be conceptualized as a content-free dimension of ideological thought reflecting the certainty with which ideological beliefs are held and the intolerance displayed towards alternative or opposing beliefs [34–36]. Evaluating the psychological similarities and differences between diverse ideological orientations in concert facilitates a comprehensive overview of the nature of ideological cognition. Here, we seek to map out the psychological landscape of these ideological orientations by investigating which psychological factors among those measured by a large battery of cognitive tasks and personality surveys are most predictive of an individual's ideological inclinations. This work aims to bridge methodologies across the cognitive and political sciences, identify key foci for future research, and illustrate the use of incorporating cognitive and personality assessments when predicting ideological convictions.

**Figure 1.**
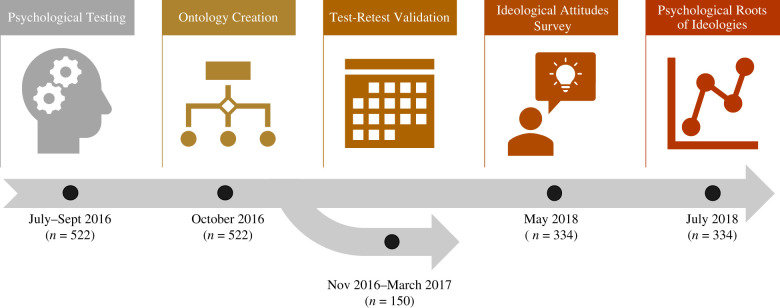
Study timeline. Collection of psychological data (37 cognitive tasks and 22 personality surveys) took place in 2016. Selection of the psychological paradigms is outlined in the Eisenberg *et al.* work [37]. The ontologies were derived [38] the test–retest reliabilities of the psychological paradigms were tested [39] (in a subsample of 150 participants) throughout 2016 and 2017. The present study reflects the last two steps in 2018, when 334 participants of the original 522 completed ideological attitudes surveys, allowing us to investigate the psychological correlates of diverse ideological attitudes.

**Figure 2.**
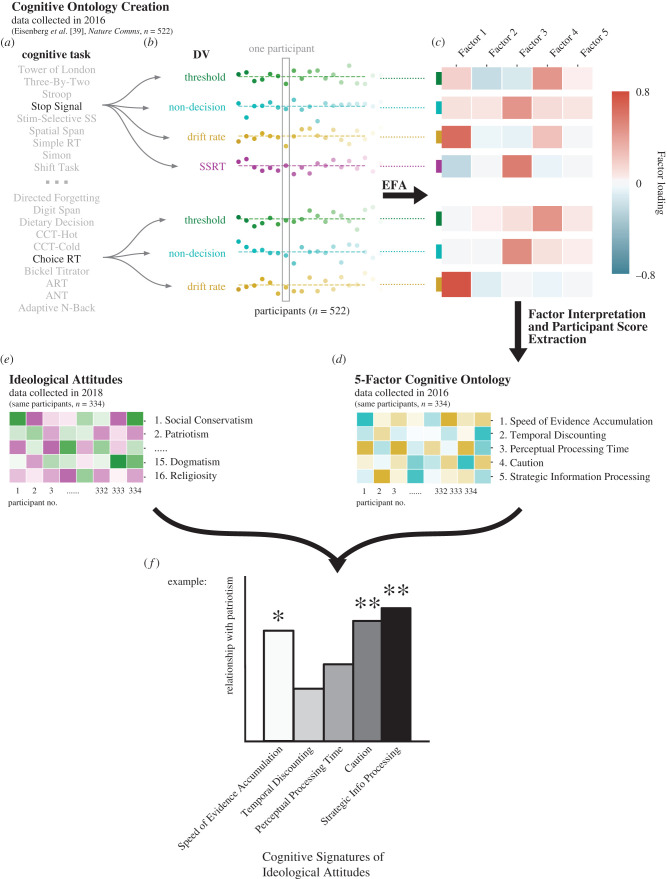
Summary of task analytic pipeline. Data-driven derivation of the cognitive task ontology (described in Eisenberg *et al*. [38] based on 522 participants allowed us to extract the ontology factor scores for the 334 participants of the current study to produce cognitive signatures of ideological attitudes. (*a*) Participants completed 37 separate cognitive task measures, of which a subset are shown. (*b*) First-level analysis of each measure resulted in a number of dependent variables (DVs). Choice Reaction Time and Stop Signal are shown as two example measures, from which seven DVs are extracted by means of drift-diffusion modelling (DDM). Participant scores are displayed as deviations from the mean for each of the seven DVs. A subset of the 522 total original participants are shown as individual dots. (*c*) Exploratory factor analysis (EFA) projects each DV from a 522-dimensional participant feature space to a lower-dimensional factor feature space. (*d*) Five factors emerged from the EFA on the cognitive data. (*e*) Three hundred and thirty four participants of the 522 original participants completed ideological attitudes surveys, facilitating (*f*) analysis of the relationships between participants' ideological attitudes and cognitive dispositions. Colour gradient in (*d*) and (*e*) reflects the participants’ scores on the factors. Adapted with permission [38].

The current study builds on recent work by Eisenberg *et al.* [37,38], in which a large sample of participants (*n* = 522) completed an extensive set of 37 well-established cognitive tasks and 22 self-report surveys focused on self-regulation and personality characteristics. The process of selecting these measures from the relevant literatures was described in detail by Eisenberg *et al.* [37], but importantly, this was completed prior to and with no relation to the question of ideologies (figure 1). Through factor analysis, Eisenberg *et al.* [38] constructed data-driven *ontologies* of cognition and personality, identifying a 5-factor structure for the cognitive task variables and a 12-factor structure for the personality survey variables. The power of these ontologies to predict real-world health outcomes was evaluated [38]. A study of test–retest reliabilities demonstrated that the ontology factor scores possessed high stability over time [38,39] (four-month mean test–retest reliability across factors of cognitive task ontology: *M* = 0.82; personality survey ontology: *M* = 0.86; *n* = 150); this reliability helps to address the challenges of obtaining robust individual differences from cognitive paradigms [39–41]. In the present investigation, we successfully recruited 334 participants (49.4% female; age: *M* = 37.07, s.d. = 8.49, range = 22–63, all United States (US) residents) from Eisenberg *et al.*'s original sample [37] and administered surveys pertaining to various political, nationalistic and religious ideological beliefs, as well as dogmatism and its conceptual inverse, intellectual humility (figure 2). This allowed us to address the question: what psychological factors are most predictive of individuals’ ideological orientations?

The 5-factor cognitive ontology was created by decomposing each of the 37 cognitive tasks into multiple dependent measures that reflected psychologically meaningful variables, such as accuracy scores (e.g. in the case of the Keep Track task that requires working memory), contrasts between different task conditions (e.g. in a task-switching task, including task-switch cost and cue-switch costs) and fitted model parameters used to capture speeded decision-making processes [38]. Wherever appropriate, performance on two-choice tasks was modelled using the drift-diffusion model (DDM), which transforms accuracy and reaction time data into interpretable latent variables including *drift rate* (corresponding to the average rate of evidence accumulation), *threshold* (corresponding to response caution in terms of speed-accuracy trade-off) and *non-decision time* (corresponding to the speed of perceptual stimulus processing and motor execution). This resulted in a total of 129 dependent cognitive measures, which exploratory factor analysis and model selection based on the Bayesian information criterion (BIC) reduced to five primary cognitive factors labelled according to their strongest loading variables: (i) *Caution* (capturing the DDM threshold parameter), (ii) *Perceptual Processing Time* (capturing the DDM non-decision time parameter and stop-signal reaction times associated with response inhibition processes), (iii) *Speed of Evidence Accumulation* (capturing the DDM drift rate parameter and other related processes), (iv) *Temporal Discounting* (reflecting variables associated with the ability to delay immediate gratification for a larger future reward), and (v) *Strategic Information Processing* (reflecting variables associated with working memory capacity, planning, cognitive flexibility and other higher-order strategies occurring at a longer time-scale than the speeded decisions modelled by the DDM). Detailed information on the nature of the ontology and its constituent elements can be found in papers by Eisenberg *et al.* [37–39].

**Figure 3.**
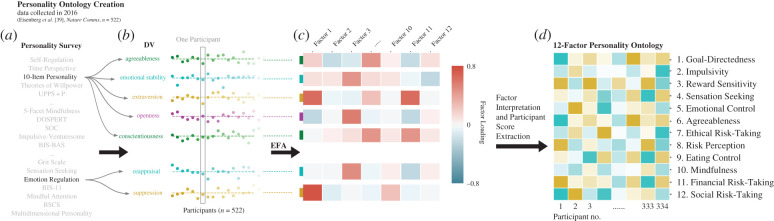
Creation of the personality ontology using (*a*) 22 personality surveys, (*b*) involving 64 separate dependent variables (DVs) that were then (*c*) subjected to exploratory factor analysis (EFA). (*d*) This revealed 12 factors, labelled in the figure. For the present study, each participant's factor scores on these 12 personality ontology factors were extracted and analysed in relation to their ideological attitudes. Adapted with permission from Eisenberg *et al.* [38].

The same methodology was applied to the 22 self-report personality surveys, resulting in 64 dependent measures that were reduced to 12 factors using oblique exploratory factor analysis (figure 3). These personality factors were associated with specific measurement scales aimed at assessing various psychological constructs, for example, Social Risk-Taking and Impulsivity. The resulting 12 personality factors were labelled based on their associated measures as indexing: (i) goal-directedness, (ii) impulsivity, (iii) reward sensitivity, (iv) sensation-seeking, (v) emotional control, (vi) agreeableness, (vii) ethical risk-taking, (viii) risk perception, (ix) eating control, (x) mindfulness, (xi) financial risk-taking, and (xii) social risk-taking. The original selection of surveys and tasks was guided by a focus on measures intended to capture self-regulation and goal-directed behaviour [37]. Notably, personality was here broadly construed in terms of self-reported psychological traits measured with established surveys that aim to tap into stable individual differences, and so personality was not defined in terms of any particular model of personality (e.g. the Big Five, though a measure of the Big Five traits was included in the creation of the survey ontology, see figure 3).

By fractionating individual differences in psychological traits into self-reported personality and behaviourally assessed cognition, we address the diversity in assessment methods used by social and cognitive psychologists to measure ‘cognitive style’ [5,17]. Indeed, recent studies have shown that self-report and behavioural measures of psychological traits may tap into different processes [37,38,42], and that the relationship between ideological leanings and cognitive style may be stronger when the latter is measured with self-report questionnaires rather than behavioural tasks [5]. A clear methodological distinction can, therefore, illuminate the relationships between psychological dispositions and ideological beliefs.

We measured participants' ideological inclinations across multiple domains by administering 16 established surveys of ideological orientations, which were selected for inclusion following a literature review [43] that examined constructs across social and political psychology and prioritized constructs that were theoretically influential in the field (e.g. system justification, social dominance orientation and authoritarianism [44,45]), widely used and have undergone extensive scale validation (e.g. intellectual humility [46] and the social and economic conservatism scale [47]). Decisions regarding controversial or conceptually overlapping ideological measures had to be taken on balance, and led, for example, to the assessment of authoritarianism but not right-wing authoritarianism (which has been criticized for its conflation with fundamentalism or conservatism, e.g. [48–51].

As depicted in figure 1, participants completed the ideological attitudes battery approximately 25 months after the initial psychological assessment. The initial assessments did not contain measures directly pertaining to ideological attitudes. The ideological attitudes surveys included self-reported questionnaires on nationalism, patriotism, social and economic conservatism, system justification, dogmatism, openness to revising one's viewpoints and engagement with religion (see Materials and methods; the electronic supplementary material tables S1 and S2 and figure S1). Exploratory factor analysis was conducted to reduce the dimensionality of these ideological orientations, revealing a 3-factor structure corresponding to the following ideological factors: political conservatism, religiosity and dogmatism. We used the factor scores of each participant from this exploratory factor analysis to validate and condense the findings obtained via the 16 ideological orientations (see Methods and materials; electronic supplementary material, figure S4 and table S3). For the sake of brevity and clarity, the focus of the analysis is on these ideological factor scores, but the analyses and data for the constituent ideological orientations are available as well in the electronic supplementary material.

A multitude of analytic strategies were employed with the aim of rigorously testing the relationships between cognition, personality and ideology. This involved frequentist regression analyses and dimensionality reduction, as well as Bayesian modelling and Bayesian Model Averaging in order to quantify the evidential strength for the contribution of the cognitive and personality traits. This allowed us to elucidate which psychological traits were most strongly tied to the diverse ideologies examined, and to construct robust signatures and predictive models that can be used by researchers in both the cognitive and political sciences to move the field forward towards more informed theories of what makes a mind ideological.

## 2. Material and methods

### (a) Participant recruitment and demographic characteristics

Participants were recruited from an existing pool of participants who completed a wide range of cognitive tasks and surveys for Eisenberg *et al.* [37] on Amazon Mechanical Turk (MTurk). All 522 original participants were contacted via MTurk and invited to participate in an additional study for financial compensation ($7 for 30–45 min), and 334 participants completed the study. Participants completed the survey on Qualtrics. The study received ethical approval from the institution. All data and analysis code are openly available at doi:10.5281/zenodo.4434725.

With respect to demographic characteristics, participants were asked to indicate age (year of birth), gender (male, female and prefer not to say or other), educational attainment (less than high school degree, high school graduate, some college but no degree, Associate degree in college (2-year), Bachelor's degree in college (4-year), Master's degree, Doctoral degree or professional degree (JD, MD)) and income (<$10 k, $10–29 k, $30–49 k, $50–99 k, $100–199 k, $200–249 k, >$250 k, prefer not to say). Other demographic factors such as household size, residence type, ethnicity and US State residence were also collected (see the electronic supplementary material, table S4).

### (b) Ideological questionnaires

Sixteen ideological questionnaires were administered to each participant, as seen in table 1.

**Table 1.** Measures of ideological orientations.

measure	scale details (all measures were assessed on a 7-point Likert-scale from ‘strongly disagree’ to ‘strongly agree’, unless otherwise specified)
social conservatism [47]	7-item scale. Participants indicate their warmth towards a set of policies. Policies: abortion, traditional marriage, traditional values, family unit, religion, patriotism, military and national security. Scale of 0-100 with intervals of 10
economic conservatism [47]	5-item scale. Participants indicate their warmth towards a set of policies. Policies: limited government, fiscal responsibility, welfare benefits, business, gun ownership. Scale of 0-100 with intervals of 10
nationalism [52]	9-item scale. Participants rate their agreement with statements such as ‘The United States is no more superior than any other country’ (reverse-coded) and ‘We should do anything necessary to increase the power of our country, even if it means war’
patriotism [53]	9-item scale. Participants rate their agreement with statements such as ‘I find the sight of the American flag very moving’ and ‘I have great love for my country’
authoritarianism [48]	4-item scale. Participants indicate whether they believe children ought to be ‘obedient’, ‘respectful’, and ‘well-mannered’ or ‘curious’, ‘independent’, and ‘self-reliant’
social dominance orientation [54]	4-item scale. Participants rate their agreement with statements such as ‘we should not push for group equality’ and ‘superior groups should dominate inferior groups’. Scale of 0-100 with intervals of 10
system justification [55]	8-item scale. Participants are presented with statements such as ‘In general, American society is fair’ and ‘American society is set up so that people usually get what they deserve’
extreme pro-group actions [56]	5-item scale. Participants are asked to rate their agreement with statements such as ‘I would fight someone insulting or making fun of America as a whole’ and ‘I would sacrifice my life if it saved another American's life’
dogmatism [57]	11-item updated version of Altemeyer's [58] measure of dogmatism
intellectual humility [46]	Comprehensive Intellectual Humility Scale measuring four facets of intellectual humility: Factor 1: independence of intellect and ego Factor 2: openness to revising one's viewpoint Factor 3: respect for others’ viewpoints Factor 4: lack of intellectual overconfidence
importance of religion (Pew Research Centre)	participants were asked: ‘How important is religion in your life?’ Response options: not at all important, slightly important, moderately important, very important, extremely important
religious prayer frequency (Pew Research Centre)	participants were asked: ‘People practice their religion in different ways. Outside of attending religious services, how often do you pray?’ Response options: several times a day, once a day, a few times a week, once a week, a few times a month, seldom, never
religious service attendance frequency (Pew Research Centre)	participants were asked: ‘Aside from weddings and funerals, how often do you attend religious services?’ Response options: more than once a week, once a week, once or twice a month, a few times a year, seldom, never

### (c) Exploratory factor analysis

To reduce the dimensionality of the ideological orientations, exploratory factor analysis using oblimin rotation was conducted using the ‘fa’ function from the R package *psych* [59]. Scree plots and parallel analysis both suggested a 3-factor structure was the most appropriate reduction of the data (see the electronic supplementary material, figure S4). The moderate correlations between the three ideological factors suggested that they reflected largely independent constructs (see the electronic supplementary material, table S2).

### (d) Cross-validation method

Cross-validated prediction of ideological outcomes was performed using ridge regression and employing a balanced 10-fold procedure (custom code based on [38,60,61]; for useful primer see [62]). This analysis divides the sample into 10 groups and fits the model on nine-tenths of the participants and tests the model on the left-over one-tenth of the sample. Across all folds each participant's ideological characteristics were predicted in a cross-validated manner, resulting in out-of-sample estimates for each participant's ideological scores. The *R*^2^ was thus computed through 10-fold cross-validated ridge regression using the RidgeCV function from scikit-learn with default parameters.

One potential (though unlikely) issue with our prediction analysis is the possibility of data-bleeding between cross-validation folds as a result of the factor analytic models. That is, the cognitive and personality ontologies were derived based on the 522 person sample collected by Eisenberg *et al.* [38]. This data-bleeding could inappropriately inflate prediction estimates. To control for this possibility we created an empirical null distribution of prediction success by shuffling the ideological outcomes and repeating the prediction 2500 times. The top 95% of this shuffled prediction success was used as a significance cut-off (*p* < 0.05).

## 3. Results

**Figure 4.**
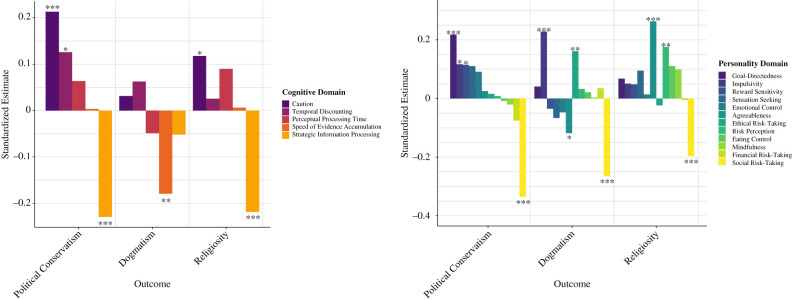
Standardized estimates of the cognitive and personality variables for each ideological factor. *p < 0.05, **p < 0.01, ***p < 0.001.

In order to understand the cognitive and personality bases of these ideological orientations, we computed a series of multiple regression analyses on each of the 16 measured ideological orientations, as well as the three summative ideological factors. Two linear multiple regression analyses were conducted for each ideological outcome variable, whereby each analysis consisted of regressors associated with one of the following feature matrices: (i) 5-factor cognitive ontology, (ii) the 12-factor personality ontology. We used the standardized beta coefficients of the linear regression models to generate a ‘cognitive signature’ and ‘personality signature’ of each ideological orientation. Figure 4 depicts the standardized estimates of the cognitive and personality ontology scores for each of the three summative ideological factors (see the electronic supplementary material, figures S5–S8 for the psychological signatures of all the ideological orientations).

**Figure 5.**
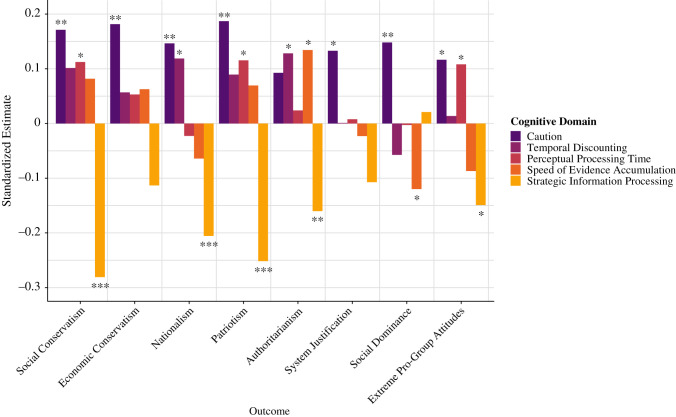
Standardized estimates of cognitive variables for ideological orientations that load on the political conservatism factor. *p < 0.05, **p < 0.01, ***p < 0.001.

The results reveal both diversity and specificity in the psychological correlates of political conservatism, dogmatism and religiosity. The political conservatism factor, which reflects tendencies towards political conservatism and nationalism, was significantly associated with greater caution and temporal discounting and reduced strategic information processing in the cognitive domain, and by greater goal-directedness, impulsivity, and reward sensitivity, and reduced social risk-taking in the personality domain. As an illustration, figure 5 demonstrates the cognitive correlates of all the ideological orientations captured by the political conservatism factor, revealing that the conservative-leaning political ideologies were consistently related to greater caution on speeded tasks and reduced strategic information processing, with some variability in the role of temporal discounting, perceptual processing time and speed of evidence accumulation. The dogmatism factor was significantly associated with reduced speed of evidence accumulation in the cognitive domain and by reduced social risk-taking and agreeableness as well as heightened impulsivity and ethical risk-taking in the personality domain. Similarly to political conservatism, the religiosity factor was also significantly associated with greater caution on speeded tasks, and reduced strategic information processing and social risk-taking, but in contrast to dogmatism and political conservatism, religiosity was associated with greater agreeableness and risk perception.

**Figure 6.**
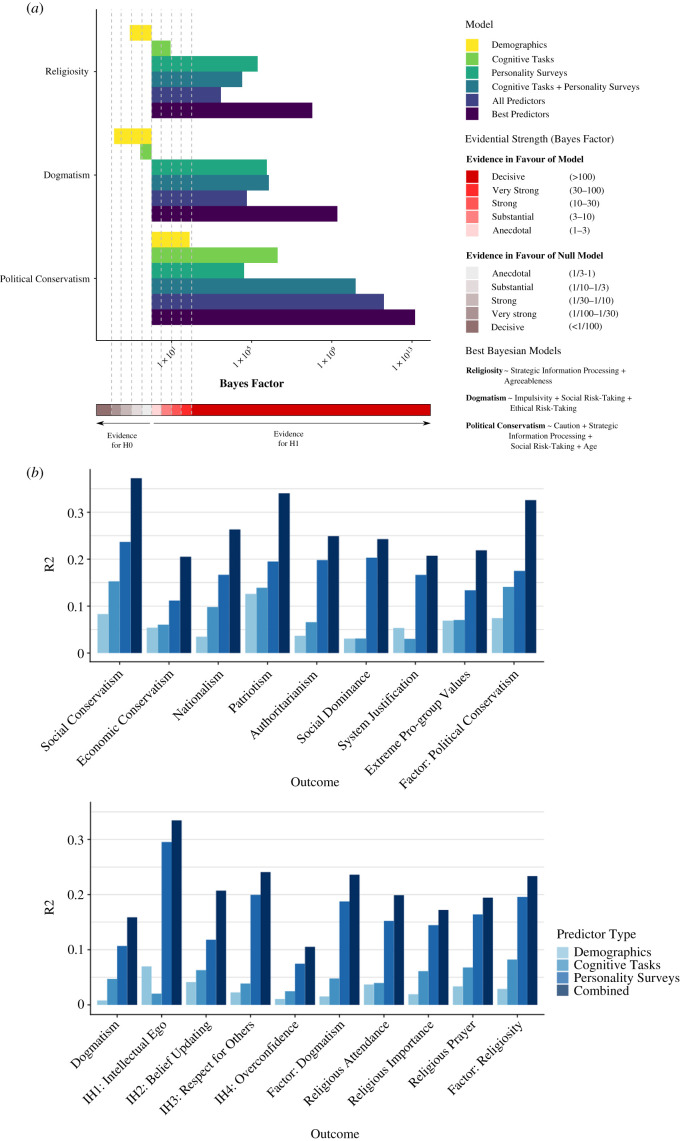
(*a*) Bayes factors for the three ideological factors for six regression models according to the model type, relative to intercept-only null hypothesis models (BF_10_). The ‘best’ models in terms of Bayes factors are shown. Evidential strength guidelines follow the classification scheme offered by Jeffreys [63] and advocated by Wetzels *et al.* [64]. For clarity, the *x*-axis is presented on a logarithmic scale. (*b*) Association of ideological orientations with demographic variables, cognitive task variables, personality survey variables, and all variables combined. Linear regression *R*^2^ are shown according to predictor type. The outcome variables are arranged according to the three ideological factors derived using exploratory factor analysis: political conservatism (top panel), dogmatism (bottom panel) and religiosity (bottom panel).

Next, we investigated the relative roles of demographic variables, self-reported personality and cognition to ideological attitudes. As evident in figure 6*b*, for the political conservatism factor, demographic variables alone explained 7.43% of the variance, while demographics and the psychological variables together explained 32.5% of the variance (4.4-fold increase). For the religiosity factor and the dogmatism factor, demographics explained 2.90% and 1.53% of the variance, respectively, while the combined model explained 23.35% and 23.60% of the variance, respectively (corresponding to an 8-fold and 15-fold increase, respectively). Consequently, including the cognitive and personality variables led to a considerable increase in the explanatory power of these models.

To further examine the evidential strength for the roles of demographic variables, self-reported personality and behaviourally assessed cognition to the three ideological attitude factors, we computed Bayes factors, which express the relative likelihood of two regression models given the data and prior expectations. To calculate Bayes factors using Bayesian regression, we relied on a default Bayesian approach promoted by Wetzels *et al.* [64], Rouder & Morey [65] and Liang *et al*. [66], and computationally specified in the R package BayesFactor [67] (using the default Cauchy priors). We computed Bayes factors, relative to the null hypothesis (BF_10_), for the regression models consisting of the different predictor types: (i) demographic variables (age, gender, educational attainment and income), (ii) cognitive ontology, (iii) personality ontology, (iv) the psychological variables (i.e. the cognitive and personality ontologies combined), and (v) the combined demographic and psychological variables. Finally, models containing the ‘best predictors’ out of the combined variable set were built using Bayesian Model Averaging, as described below.

As evident in figure 6*a*, there was decisive evidence for all models consisting of both cognitive and personality variables. The demographics-only regression model was substantially more likely than a null model given the present data for the political conservatism factor (BF_10_ = 78.26) but there was strong evidence in favour of the null model for the dogmatism factor (BF_10_ = 0.01354) and the religiosity factor (BF_10_ = 0.081655; figure 6*a*). This suggests that demographic variables play a key role in explaining ideological attitudes in the realm of politics, but do not explain religiosity or dogmatism in the current dataset.

The Bayes factor analysis further illustrates that there is substantial evidence in favour of the role of cognition in religiosity, and decisive evidence in favour of its role in political ideology. By contrast, there is anecdotal evidence in favour of the null hypothesis model relative to a cognition-only model in the case of dogmatism, suggesting that adding cognitive features does not provide added explanatory power over the intercept-only model after taking into account additional model complexity. Across all three ideological factors, there is decisive evidence in the current data in favour of the role of personality variables, as well as for models predicted by both personality and cognition, and for a combined model with all the psychological and demographic variables. In line with past research [5], the personality survey ontology was more predictive of ideological attitudes than the cognitive task ontology (figure 6); an effect that was more pronounced for dogmatism and religiosity than political conservatism, highlighting the importance of both measurement types.

Additionally, to evaluate the strength of the evidence for the psychological models (containing cognitive and personality regressors) relative to a model based solely on demographic variables, we also computed Bayes factors for all the regression models relative to the demographic-only model (BF_1D_; see electronic supplementary material, figure S9). This corroborated the findings obtained using the BF_10_, as the data was extremely more likely to occur under models containing only cognitive and personality variables than a demographics-only model (political conservatism factor: BF_1D_ = 1.975 × 10^8^; dogmatism factor: BF_1D_ = 5.248 × 10^7^; religiosity factor: BF_1D_ = 3.345 × 10^5^).

To assess the predictive power of these variables, we performed an out-of-sample prediction using 10-fold cross-validation with L2-regularized linear regression to predict participants' ideological orientations and ideological factor scores using the cognitive and personality ontologies. This contrasts with normal in-sample linear regression, which involves identical models but which are fit on the whole dataset and then fit to the same dataset, rather than to a different dataset or a subset of the data. Conducting out-of-sample cross-validation thus helps avoid problems of overfitting and is a more genuine measurement of ‘prediction’ than standard regression methods (e.g. [68]). As evident in electronic supplementary material, figure S10, the cross-validated findings were consistent with the in-sample linear multiple regression findings; the cognitive and personality ontologies were significantly predictive of participants' ideological attitudes.

**Figure 7.**
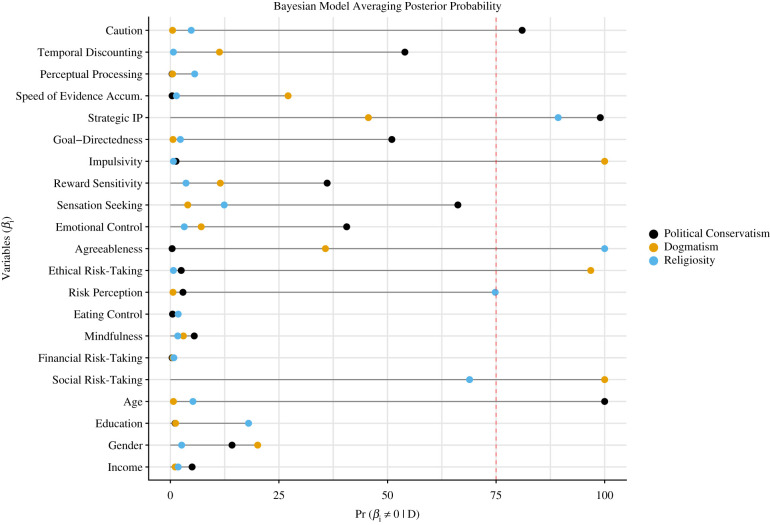
Posterior probability that each variable (*β*_i_) is non-zero given the data, D, (in %) following Bayesian Model Averaging on each of the three ideological factors. Selected variables for the ‘best’ Bayesian regression possessed a posterior probability above 75% (red dotted line). Variables are divided according to measurement type: top five variables represent the cognitive task ontology, the next 12 variables represent the personality survey ontology and the last four variables represent the demographic variables. All variables were included in a simultaneous regression for Bayesian Model Averaging.

We further sought to identify the ‘best’ model for each of the three ideological factors using a Bayesian Model Averaging approach (implemented in the bic.glm function in the bma R package [69]) for all possible linear additive models using the cognitive task variables, personality survey variables and demographic variables (age, gender, educational attainment and income) as regressors. The bic.glm function fits generalized linear models with the ‘leaps and bounds’ algorithm and the BIC approximation to Bayes factors [69]. In Bayesian Model Averaging, inference about each variable is based on the averaging of posterior distributions of all considered models—rather than a single selected model—given the present data (see the electronic supplementary material, figure S11 for all included models in the Bayesian Model Averaging). We used a Gaussian error distribution and defined selected variables as having a posterior probability above 75% in line with past guidelines [63,70]. For each of the three ideological factors, we then obtained the Bayes factors for the regression model composed of these selected variables. This approach excludes unnecessary predictors and allows us to generate the Bayesian regression that exhibits the best combination of fit and parsimony. As depicted in figures 6 and 7, each ideological factor was best predicted by a different set of variables, all of which were consistent with the results of the standardized estimates from the multiple linear regression (figure 4). These ‘best’ models all possessed the highest level of evidential strength relative to an intercept-only null model (BF_10_) and relative to a demographics-only (BF_1D_) model (Political Conservatism: BF_10_ = 1.428 × 10^13^, BF_1D_ = 1.825 × 10^11^; Dogmatism: BF_10_ = 1.877 × 10^9^, BF_1D_ = 1.386×10^11^; Religiosity: BF_10_ = 1.049 × 10^8^, BF_1D_ = 1.285 × 10^9^).

## 4. Discussion

While the field of political psychology has expanded and flourished over the past two decades, to the best of our knowledge there has been no data-driven and well-powered analysis of the contribution of a large set of psychological traits to a wide array of ideological beliefs. By administering an unprecedented number of cognitive tasks and personality surveys and employing a data-driven mental ontology [37,38], we were able to evaluate the relationships between individuals' cognition and personality and their ideological inclinations. This data-driven approach revealed striking parallels between individuals’ low-level cognitive dispositions and their high-level political, social and dogmatic attitudes.

The examination of a range of ideological attitudes pertaining to politics, nationalism, religion and dogmatism exposed remarkable similarities and differences between the psychological correlates of diverse ideological orientations, demonstrating that there may be core psychological underpinnings of ideological thinking across domains (such as the consistent roles of strategic information processing and social risk-taking; figures 4, 5 and 7, and electronic supplementary material, figures S5–S8) as well as specificity that depends on the content of the ideological domain (such as the differing contributions of caution, evidence accumulation rate, impulsivity and agreeableness). Bayesian analysis highlighted that the most parsimonious and predictive models of political conservatism include both behaviourally assessed cognitive variables and self-reported personality variables (figures 4, 6 and 7), suggesting that both measurement types are valuable for predicting ideological behaviour and should be treated as complementary sources of explained variance.

Dogmatic participants were slower to accumulate evidence in speeded decision-making tasks but were also more impulsive and willing to take ethical risks (figure 4 and electronic supplementary material, figure S6). This combination of traits—impulsivity in conjunction with slow and impaired accumulation of evidence from the decision environment—may result in the dogmatic tendency to discard evidence prematurely and to resist belief updating in light of new information. This psychological signature is novel and should inspire further research on the effect of dogmatism on perceptual decision-making processes. It is noteworthy that impulsivity differs here from caution (implicated in political conservatism and religiosity) in terms of measurement method (self-report survey versus behavioural task) and its relationship to self-control: caution here is operationalized as a trade-off between speed and accuracy under conditions where both are emphasized and so is under the influence of some strategic control, whereas impulsivity can be conceptualized as a deficit in inhibitory control rather than a strategic trade-off [71]. Consequently, dogmatic individuals may possess reduced inhibition that could be compounded by slower information uptake, leading to impulsive decisions based on imperfectly processed evidence. There has been remarkably little contemporary research on the cognitive basis of dogmatism, with a few exceptions [17–19,72,73], and so we hope these findings will stimulate further in-depth research on the perceptual underpinnings of dogmatic thinking styles.

Political conservatism was best explained by reduced strategic information processing, heightened response caution in perceptual decision-making paradigms, and an aversion to social risk-taking (figures 4, 5 and 7). These three predictors were consistently implicated in the general political conservatism factor (figure 4), as well as the specific political-ideological orientations studied, such as nationalism, authoritarianism and social conservatism (figure 5 and electronic supplementary material, figure S5). These data-driven findings are remarkably congruent with existing theoretical and empirical accounts within political psychology and also add important insights. Firstly, the finding that political and nationalistic conservatism is associated with reduced strategic information processing (reflecting variables associated with working memory capacity, planning, cognitive flexibility and other higher-order strategies) is consistent with a large body of literature [2,5] indicating that right-wing ideologies are frequently associated with reduced analytical thinking [74,75] and cognitive flexibility [6,15,17]. Additionally, conservative political ideology was characterized by a diminished tendency to take social risks (figure 4 and electronic supplementary material, figure S4) such as disagreeing with authority, starting a new career mid-life and speaking publicly about a controversial topic. This corroborates research showing that political conservatives tend to emphasize values of conformity, ingroup loyalty and traditionalism [76–80]. These empirical consistencies between the current data-driven findings and past theory-motivated research endow the present line of work with further credibility.

A politically conservative outlook was associated with greater caution in ideologically neutral speeded decision-making tasks, as operationalized in terms of the DDM parameter for the amount of evidence required before committing to a decision. Specifically, the caution with which individuals process and respond to politically neutral information was related to the conservatism with which they evaluate socio-political information (figures 4 and 5). It, therefore, appears that caution may be a time-scale independent decision strategy: individuals who are politically conservative may be perceptually cautious as well. This finding supports the idea of ‘elective affinities’ [1] between cognitive dispositions and ideological inclinations and is compatible with the perspective that political conservatism is associated with heightened motivations to satisfy dispositional needs for certainty and security [2,3,81,82]. Nonetheless, to the best of our knowledge, ideological attitudes have never before been investigated in relation to caution as measured with cognitive tasks and drift-diffusion parameters. The present results, therefore, offer a novel addition to this literature by suggesting that political conservatism may be a manifestation of a cautious strategy in processing and responding to information that is both time-invariant and ideologically neutral, and can be manifest even in rapid perceptual decision-making processes. This is relevant to the wealth of novel research on the role of uncertainty in the neural underpinnings of political processes [26,27,31,83].

The findings reveal further unexplored dynamics by highlighting that ideological orientations which have been widely studied and debated in political psychology exhibit both uniformity and variability in their cognitive and personality predictors. For example, although social and economic conservatism possessed many overlapping correlates (such as heightened goal-directedness and caution; figure 5 and electronic supplementary material, figure S5), economic conservatism was associated with enhanced sensation-seeking, whereas social conservatism was not, and in turn, social conservatism was related to heightened agreeableness and risk perception, while economic conservatism was not (electronic supplementary material, figure S5). This bears on recent debates regarding the need to fractionate conservatism into its social and economic components in order to effectively and comprehensively understand its psychological underpinnings [17,43,84–87], and highlights sensation-seeking and risk perception as potential candidates for future study. The results can also help to disambiguate past debates about the conceptual overlaps between ideological orientations such as social dominance orientation, system justification and authoritarianism [44] and their differential predictive power in relation to real-world outcomes such as prejudice [88–90] and policy attitudes [91]. Here, we found that each of these ideologies exhibited a different cognitive and personality signature.

The psychological signature of religiosity consisted of heightened caution and reduced strategic information processing in the cognitive domain (similarly to conservatism), and enhanced agreeableness, risk perception and aversion to social risk-taking, in the personality domain (figure 4 and electronic supplementary material, figure S6). The finding that religious participants exhibited elevated caution and risk perception is particularly informative to researchers investigating the theory that threat, risk and disgust sensitivity are linked to moral and religious convictions [92–97], and that these cognitive and emotional biases may have played a role in the cultural origins of large-scale organized religions [98,99]. The results support the notion that experiencing risks as more salient and probable may facilitate devotion to religious ideologies that offer explanations of these risks (by supernatural accounts) and ways to mitigate them (via religious devotion and communities).

The present data-driven analysis reveals the ways in which perceptual decision-making strategies can percolate into high-level ideological beliefs, suggesting that a dissection of the cognitive anatomy of ideologies is a productive and illuminating endeavour. It elucidates both the cognitive vulnerabilities to toxic ideologies as well as the traits that make individuals more intellectually humble, receptive to evidence and ultimately resilient to extremist rhetoric. Interestingly, the psychological profile of individuals who endorsed extreme pro-group actions, such as ideologically motivated violence against outgroups, was a mix of the political conservatism signature and the dogmatism signature (figure 5 and electronic supplementary material, figure S5). This may offer key insights for nuanced educational programmes aimed at fostering humility and social understanding [100]. By adopting research practices such as relying on comprehensive measurement approaches, integrating assessment methods from cognitive and social psychology, using both frequentist and Bayesian analytic techniques, and temporally separating the collection of psychological and ideological data, the current investigation was able to overcome many methodological concerns in social and political psychology regarding biased hypothesis generation and reproducibility [8]. The convergence between these data-driven results and past theory-driven research helps to validate existing findings and to highlight the degree to which human ideological inclinations are rooted in cognitive dispositions. Moreover, this data-driven approach generated notable novel insights that will help guide future research, such as the role of evidence accumulation rates and impulsivity in dogmatism, or the manifest relationship between political conservatism and cognitive caution in speeded perceptual decisions (figures 4 and 5). These findings underscore the fruitfulness of examining the relationships between high-level ideological attitudes and low-level cognitive processes, and suggest that ideological beliefs are amenable to careful cognitive and computational analysis [20,101]. Additionally, the results support predictive models of ideological orientations that incorporate cognitive and personality factors (figures 4, 6 and 7), carving the way for more interdisciplinary dialogue in terms of psychological methodology. Future cumulative research will need to elucidate the question of causality and translate these findings to more diverse and representative samples [102] that address the role of context in these relationships [103,104]. Recent accounts suggest that not only do psychological processes underlie ideological attitudes, attitudes also guide behaviour and decision-making across domains in ways that can shape perception, cognition and personality [6,33,105]. A wholistic, domain-general approach to the relationship between ideology and cognition can, therefore, offer a valuable foundation for research on the psychological roots of intergroup attitudes, xenophobia and ideological extremism—illustrating the myriad ways in which subtle variations in mental processes can predispose individuals to ideological worldviews.

## Data accessibility

All data, code and materials used in the analysis are available through the following links or will be made available on the Open Science Framework prior to publication: https://github.com/IanEisenberg/Self_Regulation_Ontology; https://ianeisenberg.github.io/Self_Regulation_Ontology/supplement_figures.html; https://ianeisenberg.github.io/Self_Regulation_Ontology/cleaning_visualization.html.
